# Emotional responding to overt and subtle social exclusion among young women who engage in non-suicidal self-injury

**DOI:** 10.1098/rsos.221100

**Published:** 2023-03-08

**Authors:** Kealagh Robinson, Mark E. Boyes, Marc S. Wilson, Gina M. Grimshaw

**Affiliations:** ^1^ School of Population Health, Faculty of Health Sciences, Curtin University, Perth, Australia; ^2^ enAble Institute, Faculty of Health Sciences, Curtin University, Perth, Australia; ^3^ School of Psychology, Victoria University of Wellington, Wellington, New Zealand

**Keywords:** self-harm, emotion dysregulation, social rejection, non-suicidal self-injury

## Abstract

People who engage in non-suicidal self-injury (NSSI) consistently report greater emotion reactivity and dysregulation than their peers. However, evidence that these self-reports reflect an amplified emotional response under controlled conditions is limited. Here we test the effects of both subtle and overt social exclusion, to determine whether self-reported emotion dysregulation reflects responses to real-time emotional challenge for people who self-injure. We recruited 100 young women with past-year NSSI and 100 without NSSI to an online experiment. Participants took part in a baseline social inclusion ball-tossing game, followed by either an overt or subtle social exclusion ball-tossing game, while we measured negative mood and belongingness. Despite reporting greater emotion reactivity (*d* = 1.40) and dysregulation (*d* = 1.63) than controls, women with past-year NSSI showed no differences in negative mood or belongingness ratings in response to either overt or subtle social exclusion. Within the NSSI group, exploratory analyses found greater endorsement of intrapersonal functions predicted greater negative mood following social exclusion (*β* = 0.19). Given that amplified emotional responding is central to prominent theoretical models of NSSI, findings highlight the need to better understand the divergence in findings between self-reported emotion dysregulation and real-time emotional responding among people who self-injure.

## 1. Introduction

Non-suicidal self-injury (NSSI), the intentional and self-inflicted damage of body tissue that occurs without suicidal intent, is a significant mental health concern. NSSI is concurrently associated with mental illness (e.g. [1]) and prospectively predicts poorer psychosocial functioning (e.g. [2,3]) as well as increased suicide risk [4]. Approximately 23% of young people report having engaged in NSSI [5], typically doing so to regulate unwanted emotional experiences [6]. Prominent theoretical models of NSSI argue an amplified response to emotional challenge creates the context in which an individual chooses to regulate their emotional experiences with NSSI [7–9]. Consistent with these theoretical assertions, meta-analyses find that NSSI is robustly associated with self-reports of global emotion reactivity and dysregulation [10,11] and that affect dysregulation self-reports prospectively predict NSSI [12]. In addition, reductions in global emotion dysregulation mediate the relationship between therapeutic interventions and reductions in NSSI [13,14], highlighting emotion dysregulation as a mechanism of change. Together, self-reports of global emotional responding suggest that NSSI is a behaviour characterized by amplified emotional responses to challenge. However, these self-reports rely on an individuals' evaluation of their emotional responses, rather than directly observing this process in real time.

Studies tracking the emotional response as it unfolds among people who self-injure present a more mixed picture. Studies have found that, compared with controls, people who self-injure experienced greater negative mood following social conflict [15] and interpersonal stress [16]. Other studies have reported no difference by self-injury status in emotional responses to anger inductions [17], social stress [18] or social distress and criticism scripts [19,20]. Still, other studies have found that people who self-injure show *less* negative mood following a sad film clip [21] or after writing about personal failure [22]. Given that these mixed experimental findings appear in the context of robust group differences in global emotion functioning, identifying if, and under which circumstances, people who self-injure show an amplified emotional response is critical.

Investigating how people who self-injure appraise emotional situations may offer new insights. Emotional appraisal is a highly idiosyncratic process comprising an individual's overarching (e.g. survival) and current goals (e.g. getting to work on time), as well as their values, needs and beliefs [23,24]. Meta-analyses demonstrate that people with depression and social anxiety are more likely to interpret ambiguous stimuli as more negative and less positive compared with people without these disorders [25,26], suggesting individual differences in appraisal of neutral or benign stimuli may occur in psychopathology. Drawing from personality and social psychology theories, the Situation Strength Hypothesis suggests that the impact of personality is strongest in ‘weak’ situations with fewer implicit and/or explicit cues for socially desirable behaviours and blunted in ‘strong’ situations with more cues [27]. Applied to emotional situations, the Situation Strength Hypothesis suggests that individual differences impact the emotional response *less* in instances of strong challenge (e.g. a beloved family member dying) and *more* in instances of weak or benign challenge (e.g. waving at an acquaintance who does not respond).

Limited research has investigated emotional appraisal in NSSI. Perini *et al.* [28] assessed appraisal outcomes among adolescent women with past six-month NSSI and those with no lifetime NSSI. Participants took part in a social media interaction task where they appeared to receive positive and negative feedback about themselves from other participants (in reality, simulated participants). Although both groups received equal amounts of positive and negative feedback, the NSSI group reported experiencing rejection more often than the Control group [28]. Focusing on intentional readjustment of an initial appraisal, Davis and colleagues [29] found that adults who self-injure were less effective than controls at using reappraisal to repair their negative mood following emotional challenge, and showed greater amygdala activation while under reappraisal instructions, suggesting elevated emotional processing. Taken together, there is initial evidence that people who self-injure may be more likely to appraise ambiguous emotional challenges as negative compared with controls.

In this study, we compared how young women with past-year NSSI and those without NSSI subjectively respond to two versions of social exclusion: overt (total) exclusion, and subtle (partial) exclusion. If a generalized amplified emotional response creates the context for NSSI, then women with past-year NSSI should show a larger subjective emotional response to social exclusion than women without NSSI. We also explore how women with NSSI respond to milder, more subtle emotional challenge, to test whether the appraisal of a social situation (and thus, the subjective emotional response) differs by NSSI status. We test two pre-registered hypotheses that, relative to controls, women who self-injure will either: (i) have a greater subjective emotional response to both types of social exclusion; or (ii) have a similar subjective response to overt emotional challenge, but a greater subjective response to more subtle emotional challenge.

## 2. Method

### 2.1. Participants

Based on the difference in real-time mood by NSSI status described by Fox and colleagues ([30]; *η*^2^ = 0.07), G*Power analysis indicated a sample size of 46 (23 per NSSI condition) would be sufficient at 0.95 power to detect an effect size of Cohen's *f* = 0.27 within each social exclusion condition (total *n* = 92). However, given the mixed evidence for a difference in real-time emotional responding by NSSI status, this effect size is probably an overestimate. Therefore, we approximately doubled the sample-size suggested by power analysis.

We recruited 200 women (*M* = 18.61 years, s.d*.* = 1.15 years) from an undergraduate research pool between 24 July 2020 and 11 April 2021; 100 participants reported past-year NSSI behaviour and 100 reported no lifetime NSSI behaviour. Eligible participants were women aged 17–25 years old, fluent in English, who consented to take part in self-injury related research, and were able to use a computer mouse and keyboard, with normal (or corrected to normal) eyesight. Most participants (79.0%) identified as Pākehā/New Zealand European, 6.5% identified as Māori (indigenous people of Aotearoa New Zealand), 5.0% as Indian, 1.5% as Samoan, 3.5% as Chinese, 0.5% as Tongan and 15.5% as a non-listed ethnicity. Compared with the Control group, the NSSI group were more likely to report a mental health psychiatric diagnosis ($\chi_{1}^{2}=48.24,$ *p* *<* *0*.001, Cramer's *V* = 0.49) and were more likely to be taking prescribed medications ($\chi_{1}^{2}=13.46,$ *p* < 0.001, Cramer's *V* = 0.26). Groups did not differ by age (NSSI: *M* = 18.46, s.d. = 1.03; Control: *M* = 18.75, s.d. = 1.25; *t*_198_ = 1.79, *p* = 0.075, *d* = 0.25). Within the NSSI group, participants who took part in the Total Exclusion and Partial Exclusion conditions reported similar past-year ($\chi_{5}^{2}=1.36,$ *p* = 0.929, Cramer's *V* = 0.12) and lifetime ($\chi_{5}^{2}=3.80,$ *p* = 0.579, Cramer's *V* = 0.20) NSSI frequencies. Table 1 for further information regarding the clinical characteristics of each group.

**Table 1.** Participant clinical characteristics, separated by non-suicidal self-injury status.

variable	NSSI, % or mean (s.d.)	Control, % or mean (s.d.)
any mental health diagnosis	59.0%	12.0%
depressive disorders	52.0%	5.0%
anxiety disorders	40.0%	4.0%
eating disorders	16.0%	5.0%
trauma and stressor related disorders	12.0%	—
obsessive compulsive and related disorders	8.0%	2.0%
neurodevelopmental disorders	5.0%	—
personality disorders	4.0%	—
schizophrenia spectrum and other psychotic disorders	2.0%	—
any prescribed medication(s)	43.0%	18.0%
contraceptive	17.0%	11.0%
physical health medication (e.g. asthma)	19.0%	4.0%
antidepressant	18.0%	2.0%
anxiolytic	2.0%	—
antipsychotic	3.0%	1.0%
lifetime number of NSSI methods	5.15 (2.32)	0
lifetime NSSI frequency^a^	100%	0%
1–3	6.1%	—
4–5	11.1%	—
6–10	11.1%	—
11–20	8.1%	—
21–50	24.2%	—
50+	39.4%	—
past-year NSSI frequency	100%	0%
1–3	36.0%	—
4–5	11.0%	—
6–10	15.0%	—
11–20	20.0%	—
21–50	13.0%	—
50+	5.0%	—

*Note.* NSSI = Non-suicidal self-injury.

^a^One participant in the NSSI group did not report the lifetime frequency of their NSSI.

### 2.2. Procedure

Ethical approval was obtained from Victoria University of Wellington's Human Ethics Committee. All students in two undergraduate psychology courses were invited to complete an online screening survey hosted on SurveyMonkey. Eligible students were then invited to take part in an online experiment hosted on Qualtrics in their own time. To limit NSSI-specific demand characteristics, participants were not told (until debriefing) that recruitment was based on NSSI. After providing informed consent, participants reported their demographic information, and any health diagnoses or prescription medications.

Next, participants completed the baseline Inclusion game followed by a mood rating. Participants then took part in one of two possible social exclusion conditions: Total Exclusion or Partial Exclusion. All participants then completed a second mood rating, followed by a positive mood induction where they rated nature scenes for attractiveness and familiarity. Participants were then debriefed and provided with a list of available support services. Participants took part for course credit. Preregistered hypotheses, predictions, design and analytical plans, as well as deidentified data and analyses pipelines are available at https://osf.io/qxruh.

### 2.3. Measures

#### 2.3.1. Non-suicidal self-injury

All prospective participants completed the simplified version of the Deliberate Self-Harm Inventory (DSHI-s; [31]). Participants indicate how frequently they have engaged in 13 common NSSI behaviours on a five-point scale ranging from ‘0—Never’ to ‘4—Many times’, with an additional scale point ('1—I have thought about it') included to capture NSSI ideation. The items ‘punched oneself’ and ‘banged head’ were combined. Given the Aotearoa New Zealand context, two items ('carved words…’, ‘stuck sharp objects…') were modified to exclude tā moko (the body and face marking that is part of Māori culture) [32]. DSHI scores have demonstrated convergent validity with other self-injury measures, as well as internal consistency (*α* = 0.82) and adequate test–retest over a four-week period (*φ* = 0.68) [33]. Participants recruited to the NSSI group (*n* = 100) reported having engaged in NSSI at least once in the past year. Participants recruited to the Control group (*n* = 100) indicated that they have never engaged (or thought about engaging) in 13 common NSSI behaviours [31].

Participants who reported engaging in NSSI behaviours also reported frequency of lifetime and past-year NSSI, as well as the intrapersonal and interpersonal functions of their NSSI (assessed using a 26-item version of Inventory of Statements about Self-injury; [34]). Consistent with previous research (e.g. [34]), both the Intrapersonal (*α* = 0.76) and Interpersonal functions (*α* = 0.77) subscales showed adequate internal consistency.

#### 2.3.2. Global self-report measures

Global emotion reactivity was assessed with the 21-item Emotion Reactivity Scale (ERS; [35]). Participants responded to items such as ‘I tend to get very emotional very easily’ on a 5-point Likert scale ranging from ‘0—Not at all like me’ to ‘4—Completely like me’. Global emotion dysregulation was assessed with the 16-item brief version of the Difficulties in Emotion Regulation Scale (DERS-16; [36]). Participants respond to items such as ‘When I'm upset, I believe that I will remain that way for a long time’ on a 5-point scale from ‘1—Almost never (0–10%)’ to ‘5—Almost always (91–100%)’. Item scores were totalled to give an overall score of global emotion reactivity and dysregulation. Both the ERS (*α* = 0.96) and DERS-16 (*α* = 0.96) showed excellent internal reliability.

#### 2.3.3. Social exclusion manipulation

Social interaction was modelled using Cyberball (v. 5.4.1; [37]). At the beginning of the study, participants were told they would play two online ball-passing games with other students in their course. Before each game, a screen with a ‘loading’-style GIF was presented for 5 s below the text *‘Waiting for other players to come online’*. Before each game, participants were told their task was to mentally visualize the experience in as much detail as possible. There were 40 ball tosses in each game.

In the first game, all participants took part in an Inclusion (i.e. baseline) condition where they received 32.5% of throws from the two other ‘players’ (i.e. 13 throws, approximately a ‘fair’ distribution of all throws). In the second game, participants took part in one of two Exclusion conditions. In the Total Exclusion condition, participants experienced the standard social exclusion manipulation [37,38], where they received the ball once at the beginning of the game and never again (2.5% of throws). In the Partial Exclusion condition, participants received 15.0% of throws (i.e. six throws, considerably fewer throws than would be ‘fair’)^1^, a similar proportion as previous ambiguous Cyberball manipulations [39].

#### 2.3.4. Real-time responding

Belongingness and subjective mood ratings were assessed after each Cyberball game using visual analogue scales ranging from ‘0—Not at All’ to ‘100—Extremely’, presented on the computer screen. Three items assessed belongingness. Each item began with ‘During the [first] ball-passing game…’ and were completed by ‘…I felt poorly accepted by the other participants' (reverse-coded), ‘…I felt as though I had made a ‘connection’ or bonded with one or more of the participants', and ‘…I felt like an outsider’ (reverse-coded). Participants' responses at each time point were averaged to create overall scores of belongingness ranging from 0 to 100. These belongingness items have been used previously and are sensitive to changes in between-subject manipulations of exclusion [40]. Eleven items assessed subjective mood. Each item began with ‘Right now, I feel’ and were completed by ‘angry’, ‘sad’, ‘ashamed’, ‘irritable’, ‘frustrated’, ‘anxious’, ‘alert’, ‘relaxed’, ‘interested’, ‘happy’ and ‘confident’. Item order was randomized for each presentation and for each participant. Scores for alert, relaxed, interested, happy and confident were reverse-coded prior to analysis. Participants' responses at each time point were averaged to create overall scores of negative mood ranging from 0 to 100.

### 2.4. Data analysis

When 199 participants had completed the study, we calculated the average time in minutes it took to complete the study (*M* *=* 38.83, s.d. = 140.46). Participants whose completion time was three standard deviations above the mean (*n* = 3) were removed and replaced. One participant left 13 items (54.17%) of the negative mood and belongingness visual analogue scales blank. Given that: (i) the marker participants moved to indicate their response was automatically set at 50 (i.e. the midpoint), and (ii) the participant provided no ratings between 40 and 60, these missing values were assigned a value of 50. Little's Missing Completely at Random (MCAR) test suggested the pattern of missingness on the ERS and the DERS-16 was not MCAR, *χ*^2^(641, *n* = 200) = 751.45, *p* = 0.002. However, as only 1.46% of values were missing, following convention [41], we deemed this missingness inconsequential. Missing values were imputed using expectation-maximization.

Statistical analyses were conducted using jamovi (v. 2.2.5; [42]). Statistical significance was set at *p* < 0.050, with *p* < 0.100 considered a trend for predicted effects only. All analyses reported here were preregistered unless noted as exploratory, and all preregistered analyses are reported. Chi-squared analyses tested for group differences in medication use and clinical diagnoses, and independent *t*-tests for group differences in global emotion reactivity and dysregulation. Mixed-model ANOVAs with Phase (Inclusion, Exclusion) as a within-subjects factor and NSSI Status (NSSI, Control) and Exclusion Severity (Total Exclusion, Partial Exclusion) as between-subjects factors tested for main effects and interactions in belongingness and negative mood ratings. Exploratory hierarchical linear regression assessed whether NSSI characteristics were associated with emotional response to social exclusion.

## 3. Results

### 3.1. Self-reports of global emotional responding

Consistent with previous meta-analytic research, the NSSI group reported significantly greater global emotion reactivity (*M* = 2.39, s.d. = 0.82) than the Control group, (*M* = 1.29, s.d. = 0.76, *t*_198_ = 9.87, *p* < 0.001, *d* = 1.40). Likewise, the NSSI group reported significantly greater global emotion dysregulation (*M* = 3.40, s.d. = 0.85) than the Control group (*M* = 2.09, s.d. = 0.75, *t*_198_ = 11.55, *p* < 0.001, *d* = 1.63). Within the NSSI group, past-year NSSI frequency was positively associated with both global emotion reactivity (*r* = 0.36, *p* < 0.001) and dysregulation (*r* = 0.36, *p* < 0.001). Lifetime number of NSSI methods was unrelated to both global emotion reactivity (*r* = 0.13, *p* = 0.210) and dysregulation (*r* = 0.15, *p* = 0.146).

### 3.2. Responding to social exclusion

Table 2 provides the descriptive statistics of belongingness and negative mood ratings separated by Exclusion Severity condition and NSSI status. First, we tested if both exclusion conditions effectively created feelings of social exclusion. In terms of negative mood ratings, we found a main effect of Phase (*F*_1,196_ = 135.42, *p* < 0.001, $\eta_{p}^{2}=0.41$) and a trend towards a main effect of Exclusion Severity (*F*_1,196_ = 3.58, *p* = 0.060, *η*_p_^2^ = 0.02). Critically, there was a significant interaction between Exclusion Severity and Phase (*F*_1,196_ = 4.83, *p* = 0.029, $\eta_{p}^{2}=0.02$). Although negative mood increased (relative to baseline Inclusion) in both Total Exclusion (*t*_99_ = 9.96, *p* < 0.001, *d* = 1.00) and Partial Exclusion conditions (*t*_99_ = 6.61, *p* < 0.001, *d* = 0.66), this increase was greater for Total Exclusion (Δ*M* = 14.76, Δs.d. = 14.82) than for Partial Exclusion (Δ*M* = 10.07, Δs.d. = 15.24, *t*_198_ = 2.21, *p* = 0.029, *d* = 0.31). We found a complementary pattern of results for belongingness ratings, with a main effect of Phase (*F*_1,196_ = 667.62, *p* < 0.001, $\eta_{p}^{2}=0.77$), but not Exclusion Severity (*F*_1,196_ = 1.96, *p* = 0.163, $\eta_{p}^{2}=0.01$). Importantly, there was a significant interaction between Exclusion Severity and Phase (*F*_1,196_ = 10.96, *p* = 0.001, $\eta_{p}^{2}=0.05$). Although belongingness decreased (relative to baseline Inclusion) in both Total Exclusion (*t*_99_ = 20.47, *p* < 0.001, *d* = 2.05) and Partial Exclusion conditions (*t*_99_ = 16.19, *p* < 0.001, *d* = 1.62), this decrease was greater for Total Exclusion (Δ*M* = −48.48, Δs.d. = 23.68) than for Partial Exclusion (Δ*M* = −37.47, Δ*s.d.* = 38.83; *t*_198_ = 3.33, *p* = 0.001, *d* = 0.47). Thus, although both social exclusion conditions increased negative mood and decreased feelings of belongingness, partial exclusion from other Cyberball ‘players’ did so less effectively than total exclusion, suggesting the Partial Exclusion condition was experienced as a milder emotional challenge than the Total Exclusion condition, as intended.

**Table 2.** Average belongingness and negative mood ratings, separated by exclusion severity and non-suicidal self-injury status.

variable	Total Exclusion			Partial Exclusion		
	overall sample, mean (s.d.)	NSSI, mean (s.d.)	Control, mean (s.d.)	overall sample, mean (s.d.)	NSSI, mean (s.d.)	Control, mean (s.d.)
*belongingness ratings*						
Inclusion	66.30 (17.06)	64.67 (16.69)	67.93 (17.44)	63.71 (17.96)	66.65 (15.68)	60.77 (19.7)
Exclusion	17.82 (19.36)	16.85 (19.13)	18.79 (19.73)	26.25 (20.92)	28.57 (20.44)	23.92 (21.35)
*negative mood ratings*						
Inclusion	37.23 (16.76)	43.59 (17.73)	30.87 (13.07)	35.59 (16.74)	41.09 (17.99)	30.10 (13.45)
Exclusion	51.99 (19.56)	57.72 (20.01)	46.27 (17.48)	45.66 (17.46)	52.09 (16.46)	39.24 (16.14)

*Note.* Partial Exclusion *n* = 100, Total Exclusion *n* = 100.

Next, we tested the hypotheses that, relative to controls, people who self-injure experience either: (i) a greater emotional response to social exclusion in general; or (ii) a similar emotional response to overt social exclusion, but a greater emotional response to more subtle social exclusion. For negative mood ratings, we found a main effect of NSSI Status (*F*_1,196_ = 32.52, *p* < 0.001, $\eta_{p}^{2}=0.14$), whereby the NSSI group reported greater negative mood in general than did the Control group following both baseline Inclusion (*t*_182.54_ = 5.35, *p* < 0.001, *d* = 0.76, equal variances not assumed) and Exclusion games (*t*_198_ = 4.83, *p* < 0.001, *d* = 0.68). Counter to hypotheses, we found no evidence of interactions between NSSI Status and Phase (*F*_1,196_ = 0.02, *p* = 0.888, $\eta_{p}^{2}=0.01$), NSSI status and Exclusion Severity (*F*_1,196_ < 0.01, *p* = 0.968, $\eta_{p}^{2}=0.01$), or NSSI Status, Phase and Exclusion Severity (*F*_1,196_ = 0.54, *p* = 0.464, $\eta_{p}^{2}<0.01$). Again, we found a complementary pattern of results for belongingness ratings. We found a trend toward a main effect of NSSI on belongingness ratings (*F*_1,196_ = 3.56, *p* = 0.061, $\eta_{p}^{2}=0.02$), such that there was a trend to suggest the NSSI group felt they belonged less than the Control group during the Inclusion game (*t*_198_ = 1.86, *p* = 0.065, *d* = 0.26), with no difference in belongingness during the Exclusion game (*t*_198_ = 1.13, *p* = 0.259, *d* = 0.16). Counter to hypotheses, we found no evidence of interactions between NSSI Status and Phase (*F*_1,196_ = 0.15, *p* = 0.701, $\eta_{p}^{2}=0.01$), NSSI Status and Exclusion Severity (*F*_1,196_ = 0.41, *p* = 0.523, $\eta_{p}^{2}<0.01$), or NSSI Status, Phase, and Exclusion Severity (*F*_1,196_ < 0.001, *p* = 0.987, $\eta_{p}^{2}<0.01$) in belongingness ratings. Taken together, we found no evidence that, compared with controls, people who self-injure showed either an amplified subjective response to social exclusion in general, or an amplified subjective response to subtle social exclusion specifically.

### 3.3. Non-suicidal self-injury characteristics and real-time responding

People who self-injure show considerable variability in their NSSI characteristics, and thus group-level ‘past-year NSSI status' may obscure meaningful individual differences *among* people who self-injure in how they respond to social exclusion. Focusing on the NSSI group, we conducted exploratory hierarchical linear regressions predicting belongingness and subjective mood following social exclusion. Within the regression models, we included baseline Inclusion ratings and Exclusion Condition at Step 1, and number of lifetime NSSI methods, past-year NSSI frequency, intrapersonal functions and interpersonal functions at Step 2. Table 3 provides the model fit statistics and standardized estimates.

**Table 3.** Linear regression analysis predicting responding to social exclusion by non-suicidal self-injury characteristics.

predictors	Exclusion belongingness ratings		Exclusion negative mood	
	*β* (95% CI)	*p*	*β* (95% CI)	*p*
Step 1	*F*_2,96_ = 2.57, *p* = 0.105	Adj. *R*^2^ = 0.03	*F_2,96_ = 38.42,* *p* *< 0.001*	*Adj. R^2^ = 0.43*
baseline inclusion ratings	0.14 (−0.06, 0.34)	0.159	*0.65 (0.50, 0.80)*	*<0.001*
exclusion severity condition	−0.18 (−0.38, 0.02)	0.082	0.11 (−0.05, 0.26)	0.130
Step 2	*F*_6,92_ = 1.95, *p* = 0.080	Adj. *R*^2^ = 0.06	*F_6,92_ = 15.05,* *p < 0.001*	*Adj.* *R^2^ = 0.46*
number of lifetime NSSI methods	0.22 (−0.04, 0.49)	0.096	−0.07 (−0.27, 0.12)	0.469
past-year NSSI frequency	−0.16 (−0.41, 0.08)	0.192	0.12 (−0.07, 0.30)	0.216
intrapersonal functions	*−0.26 (−0.49, −0.03)*	*0.027*	*0.19 (0.03, 0.36)*	*0.024*
interpersonal functions	0.09 (−0.12, −0.31)	0.384	0.05 (−0.15, 0.18)	0.853

*Note.* Analyses conducted within the NSSI subsample (*n* = 100). Significant findings are in italics for clarity.

For belongingness ratings, neither Step 1 nor Step 2 of the linear regression model significantly predicted feelings of belonging following social exclusion. For negative mood, Step 1 and Step 2 of the regression model significantly predicted negative mood following social exclusion. After accounting for baseline negative mood, neither number of lifetime NSSI methods, past-year NSSI frequency, or interpersonal functions predicted negative mood following social exclusion. Notably, intrapersonal functions (*β* = 0.19) positively predicted negative mood following social exclusion, suggesting the more a participant reported engaging in NSSI to manage their internal experiences the greater their emotional response to social exclusion.

## 4. Discussion

Prominent theoretical models of NSSI argue that amplified emotional responses to challenge are central to the behaviour [7–9]. A wealth of evidence supporting this assertion comes from elevated self-reports of global emotional responding [11,12]. However, these self-reports rely on an individuals' evaluation of their emotional responses, rather than directly observing this process in real time. This study compared how young women with and without NSSI subjectively respond to overt and subtle emotional challenge to determine whether self-reported emotion dysregulation is reflected in responses to real-time emotional challenge for people who self-injure.

Meta-analysis demonstrates that total exclusion from other Cyberball ‘players’ reliably creates negative mood [43]. Extending previous research [37,39], we found participants are sensitive to the extent to which they are excluded; although both social exclusion conditions increased negative mood and decreased belongingness compared with social inclusion, the Partial Exclusion game was less effective at doing so than the Total Exclusion game. Similar to previous research (e.g. [15,44]), young women with recent NSSI reported greater negative mood *in general* compared with controls. This elevated negative mood probably reflects the co-morbidity of NSSI with psychiatric disorders characterized by negative mood (e.g. [1]).

Counter to the amplified emotional response hypothesis, both NSSI and Control groups showed a similar pattern of subjective reactivity to overt social exclusion. These findings add to the growing body of evidence demonstrating no difference by self-injury status in emotional responses to social exclusion [45,46], anger inductions [17], social stress [18] or personally relevant social distress and criticism scripts [19,20,47]. Previous research using partial Cyberball exclusion indicated that high-neuroticism (compared with low-neuroticism) participants perceived themselves as having less control in the partial exclusion condition, but not in the total exclusion condition, suggesting individual differences in appraisal are most evident in appraisals of ambiguous situations [39]. Although NSSI is positively associated with neuroticism (e.g. [48–50]), we found no evidence that women who self-injure were more likely to appraise a milder, more ambiguous social exclusion as more challenging than controls.

A growing body of research documents NSSI characteristics (e.g. lifetime frequency, number of NSSI methods) are differentially associated with psychological phenomena [51,52], suggesting that it may be useful to differentiate *among* people who self-injure. Exploratory analysis within the NSSI group revealed that, accounting for baseline negative mood and exclusion severity condition, neither lifetime number of NSSI methods, past-year NSSI frequency or interpersonal functions uniquely predicted negative mood following social exclusion. However, intrapersonal functions uniquely predicted negative mood following exclusion; the more a person reported engaging in NSSI to manage their internal experiences, the greater their subjective response to social exclusion. Given foundations of individual differences in emotional lability are thought to be set in early development well *before* NSSI onset (e.g. [53]), these exploratory correlational results suggest that among people who self-injure, those who experience amplified subjective reactivity may be more likely to self-injure to manage their internal experiences. Future research should replicate this association between intrapersonal functions and subjective reactivity across a variety of emotion manipulations.

This study has two key strengths. First, in contrast to the majority of Cyberball research (e.g. [39,46,54]), we used a within-subjects exclusion manipulation, providing greater statistical power to probe emotional responding to social exclusion and allowing us to assess for baseline group differences. Second, we test for altered subjective responding to social exclusion using a sample of young adults with recent, rather than lifetime, NSSI. However, findings should be interpreted in light of five caveats. First, we recruited only women, limiting generalizability of these findings to other genders. Second, participants were asked to recall instances of NSSI in the past year. Recall may be influenced by the length of time since the last instance of NSSI, such that more recent behaviour is more accurately recalled. Third, inspection of participants' post-exclusion belongingness ratings highlights the possibility of a floor effect—34.5% of the sample rated their perceived belongingness between 0 and 10 (possible range 0–100). Other Cyberball studies have also reported zero-inflated belongingness ratings following social exclusion (e.g. [54,55]), suggesting this issue may be widespread. The low variability in belongingness ratings makes the belongingness results difficult to interpret and may indicate that participants knew *objectively* that they were being excluded. Fourth, although the Partial Exclusion condition created smaller increases in negative mood than the Total Exclusion condition, suggesting it was a milder emotional challenge, it seems unlikely the Partial Exclusion game was truly an *ambiguous* event that could reasonably be interpreted as benign. Future research could use well-established lexical ambiguity tasks (e.g. [56]) to test if NSSI is characterized by an appraisal system more sensitive to threat. Finally, the real-world validity and personal relevance of experimentally manipulated emotional challenges such as Cyberball is limited. Future research should employ established experience sampling methods to assess emotional responding to challenges in daily life (e.g. [57]) to better understand real-time emotion dysregulation in NSSI.

## 5. Conclusions

Despite reporting considerably greater global emotion reactivity and dysregulation than those without NSSI, we found no evidence to suggest that women with past-year NSSI showed an amplified or more sensitive emotional response to social exclusion. Given that amplified emotional responding is central to prominent theoretical models of NSSI [7–9], where does this dissociation between self-reports of global emotional functioning and responses to real-time emotional challenges leave us? Perhaps experimental approaches to date have systematically failed to capture the nature of emotional challenges that people who self-injure struggle with. Or perhaps it is an individual's perceptions of their ‘emotionality’, rather than their actual emotional responses, that are critical in NSSI. To advance our understanding of emotional responding in NSSI, future research should: (i) establish the conditions (if any) under which people who self-injure show amplified emotional responses and (ii) isolate the psychological processes which underlie the experience of poorer emotional functioning among people who self-injure.

## Data Availability

The data and associated analysis code are available on OSF: https://osf.io/qxruh.

## Footnotes

1 Number of throws was decided after piloting three versions (10.0%, 15.0% and 22.5% of throws) within a small sample (*n* = 26). Given that we were looking for a subtle emotional challenge that could be interpreted differently by participants, we selected the condition that resulted in the greatest variability (i.e., the largest s.d.) in mood change (Exclusion Negative Mood—Baseline Negative Mood).
